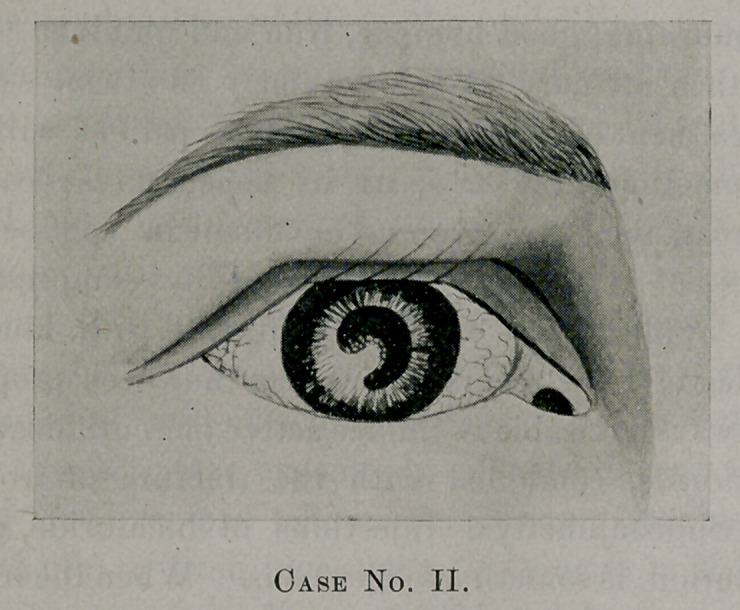# Iritis, Varieties of, Diagnosis, Differential Diagnosis, Causation, Treatment

**Published:** 1902-04

**Authors:** F. Pierce Hoover

**Affiliations:** Jacksonville, Florida, Formerly Lecturer in Otology, New York Polyclinic; Assistant Surgeon Manhattan Eye and Ear Hospital, New York City


### IRITIS, VARIETIES OF, DIAGNOSIS, DIFFERENTIAL DIAGNOSIS, CAUSATION, TREATMENT.*

BY F. PIERCE HOOVER, M.D., JACKSONVILLE, FLORIDA,

Formerly Lecturer in Otology, New York Polyclinic; Assistant Surgeon Manhattan Eye and Ear Hospital, New York City.

It is not my intention to introduce anything new regarding the management and treatment of so well-known a disease as iritis to the specialist, but I do desire to call attention of the general practitioner to how very, very important it is to recognize this disease, and distinguish it from some of the other eye affections it simulates. Those who see these cases almost daily in their practice appreciate the importance of an early diagnosis, as it may save the patient perhaps weeks and months of suffering, and possibly the loss of sight of the affected eye.

The first thing that attracts our attention to the iris when it takes on disease is hyperemia, which requires consideration as it is a feature of several other affections of the eye, viz.: Inflammation of the cornea, acute trachoma, scleritis, purulent conjunctivitis, traumatism, etc. Iritis should be divided into three groups for convenience, *i. e.*, the plastic (simple), the serous and the suppurative, the most common being the first named variety. In all cases of iritis there is pain, more or less pronounced, somewhat throbbing in character, which extends over that side of the face

*Read before the Duval County Medical Society, March 4th 1902.

and head on which the affected eye is, and, as a rule, it is much more intense at night than during the day. When pressure upon the eye-ball is made with the finger over the closed eye, the pain is increased. Objects are barely, and sometimes not, discernable; one can stand close to the patients and all they may see is a something white when the physician's face is close to theirs, while the features are unrecognizable. The pupil is sluggish and contracted, adhesions form between the iris and the lens, or it is irregular from synechia, also there is intolerance to light and a constant discharge of tears. The patient always finds that he experiences more comfort with the eyes closed, or when sitting in a dark place. The color of the iris changes and has a muddy, cloudy appearance. The iris becomes thickened and there is a zone of pericorneal redness around it. An inflammation of the iris will have a pronounced hyperemia attending it usually, but sometimes an iritis will come on *without* sufficient inflammation to attract the attention of the patient. If a case of iritis is seen late, the *entire* pupillary margin may be bound down and the pupil will *fail* to dilate under atropin or the influence of any of the mydriatics, while in the early state, only *various* portions of the pupillary margin are bound down, and pupil *will respond* to the mydriatic used by the adhesions being broken up. In the purulent type of iritis an hypopyon (pus in the anterior chamber) will make its appearance, indicating that there is present *pus*. A chronic iritis will terminate possibly in atrophy of the iris, atrophy of the bulb, or in cataract. Iritis may be either idiopathic, symptomatic or traumatic. Iritis is *most frequent* in middle life and *most rare* in childhood, and *men* are more often affected than *women*. One eye may be affected or both eyes at the same time, or one eye may take on the disease, the result of the other (sympathetic) or from inoculation. Injuries to the iris, such as a blow on the eye, wounds, contusions, result of operations on the eye for cataract, etc., or for the removal of foreign substances, such as cinders, etc., all may produce an iritis; also, iritis may be caused from result of a keratitis or choroiditis, colds, gout, syphilis, gonorrhœa, run down and anemic conditions, rheumatism, and diabetes. The *results* of iritis may be the loss of the eye, cysts, tumors, tubercle and cancer.

*Differential Diagnosis.*—"Keratitis:" the hyperemia, shows itself in a zone around the cornea as in iritis, it is deeper in color at corneal margins, growing more indistinct as the blood vessels run into the sclera. In the healthy eye these vessels are seldom seen. "Acute glaucoma:" the pupil is *dilated*, while in iritis it is *contracted*, the pain is similar to that of iritis, it has exacerbations and remissions with increased ocular tension. "Purulent conjunctivitis:" the papillæ of the conjunctiva are *very* greatly enlarged; it is from infection, generally gonorrhea, the lids are swollen and a purulent discharge sets up. "Cyclitis" rarely takes place, unless it is in connection with an inflammation of the iris or choroid, the pain is not so great as in iritis, the redness in the circumcorneal region is in a great measure like that one sees in iritis, but there is *no* iritis or keratitis to account for same. "Traumatism," we have the history of an injury as the cause of the great amount of hyperemia, etc.

*Treatment.*—If case is seen early, absolute rest, and eye kept from the light; give a dose of calomel, followed by salts or seidlitz powders next morning, good hygiene, iron and quinine. If patient is syphilitic, then mercury should regularly be given. Most important in treatment of iritis is atrophine, if patient will not tolerate it, as is sometimes the case, as its constant use causes dryness of the throat, then hyoscyamus or duboisin can be substituted; the dryness of throat can be obviated by compressing the tear duct after application. In old syphilitics with much cachexia, where a plastic iritis recurs which has not been properly treated, it is not always advisable to induce active mercurialization; for such cases bichloride, combined with the tincture of iron, is recommended. Subconjunctival injections of bichloride, gtts. 2-4 to a 1-1000 solution, is sometimes efficacious. When the iris is adherent to the capsule of the lens, it is called a posterior synechia. Frequently an eye that has had an iritis will become myopic and require glasses to correct the amount of same.

The following pen and ink drawings I recently made of two cases of iritis, both of which were the result of syphilis:

CASE I.—Patient male, 25 years old; occupation, engineer. Pupil dilated with atropin and shows the projecting synechia; there

CASE No. I.

is a general conjunctivitis, lachrymation, the pain extends to temple and right side of face, on which is the affected eye.

CASE No. II.

CASE II.—Male, salesman, 36 years; has a condylomatous iritis, showing elongated pupil, which is dilated with atropin.

*111 West Forsyth Street.*

## Figures and Tables

**Case No. I. f1:**
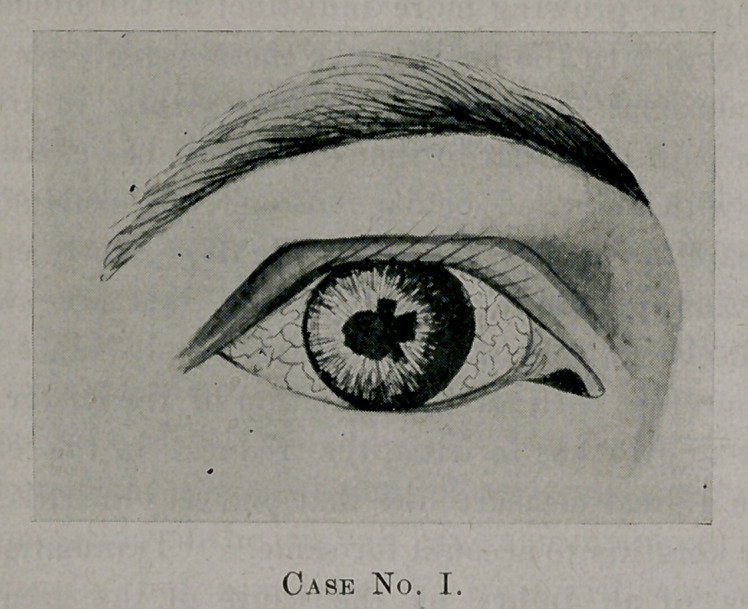


**Case No. II. f2:**